# Supplementary material for: Complexome profiling of the Chlamydomonas psb28 mutant reveals TEF5 as an early PSII assembly factor
Source: Plant Cell. 2025 Mar 17;37(6):koaf055. doi: 10.1093/plcell/koaf055 (PMC12164590; doi:10.1093/plcell/koaf055)
Supplement: koaf055_Supplementary_Data [file koaf055_supplementary_data.zip › psb28_interactive.zip!/plots/intensity/qualityPlots/peptides/_Cre12.g492350.t1.2.html]

### Cre12.g492350.t1.2

Trivial name:   
SeqCoverage [median]: 39.10  
Mass: 69.95 kDa  
  
 

| Sequence | MissedCleavage | Mass [Da] | wtIntensity | muIntensity | Score |
| --- | --- | --- | --- | --- | --- |
| AAMEAATQVGSSAVLLADRPADVTEHR | 1 | 2765.38 | 12550.68 | 15405.69 | 102.67 |
| AAPPGFGDGGVGPLAGVR | 0 | 1593.83 | 2176.77 | 0.00 | 70.44 |
| ADYPPVRPVQDSLAR | 1 | 1682.87 | 2161.15 | 0.00 | 48.14 |
| AGASGLVALLR | 0 | 1026.62 | 960.44 | 0.00 | 76.83 |
| ALAAVATGK | 0 | 800.48 | 7960.41 | 5033.85 | 82.45 |
| ASDPATPVTLPPSLR | 0 | 1520.82 | 3290.97 | 3025.97 | 81.52 |
| AVVGVIGSAHVR | 0 | 1163.68 | 4259.60 | 9947.48 | 84.48 |
| DTTVEPLLADAE | 0 | 1272.61 | 978.38 | 0.00 | 90.66 |
| FADMSADQIEDAVAINEPISGGDLSKPLK | 1 | 3030.49 | 2676.37 | 0.00 | 73.11 |
| FMSSTYAR | 0 | 961.43 | 532.73 | 706.19 | 94.26 |
| MFIPSETLEAMR | 0 | 1423.68 | 2603.90 | 2483.48 | 71.35 |
| MGATAAAAASTDGEAESASASGR | 0 | 2038.89 | 1990.27 | 376.23 | 94.38 |
| NAVAVLEAPNPTAPGGK | 0 | 1604.85 | 6630.96 | 8911.57 | 191.53 |
| SGLLVDPEDAATAPK | 0 | 1482.76 | 10386.26 | 3232.62 | 105.94 |
| SLPALTGLSAR | 0 | 1084.62 | 4203.79 | 2166.26 | 95.36 |
| TSVYLLAMSHVSK | 0 | 1434.75 | 691.02 | 0.00 | 46.06 |
| VGSVAHAR | 0 | 795.44 | 431.83 | 0.00 | 79.47 |
| YAMPEAGPAR | 0 | 1061.50 | 2718.68 | 0.00 | 90.66 |
|  |  |  | 67204.2 | 51289.4 |  |
